# Synaptosomal-associated protein 25 (Snap-25) gene Polymorphism frequency in fibromyalgia syndrome and relationship with clinical symptoms

**DOI:** 10.1186/1471-2474-15-191

**Published:** 2014-05-31

**Authors:** Ayse Balkarli, Cem Sengül, Emre Tepeli, Huseyin Balkarli, Veli Cobankara

**Affiliations:** 1 Department of Internal Medicine, Division of Rheumatology, Pamukkale University Hospital, Kınıklı, 20070 Denizli, Turkey; 2 Department of Psychiatry, Pamukkale University Hospital, Kınıklı, 20070 Denizli, Turkey; 3 Department of Medical Biology, Pamukkale University Hospital, Kınıklı, 20070 Denizli, Turkey; 4 Department of Orthopedics and Traumatology, Akdeniz University Hospital, Dumlupınar Avenue, 07070 Antalya, Turkey

**Keywords:** Fibromyalgia, Synaptosomal-associated protein 25 gene polymorphism

## Abstract

**Background:**

SNAP-25 protein is contributory to plasma membrane and synaptic vesicle fusions that are critical points in neurotransmission. SNAP-25 gene is associated with behavioral symptoms, personality and psychological disorders. In addition, SNAP-25 protein can be related to different neurotransmitter functions due to its association with vesicle membrane transition and fusion. This is important because neurologic, cognitive, and psychologic disorders in fibromyalgia syndrome (FMS) can be related to this function. This relationship may be enlightening for etiopathogenesis of FMS and treatment approaches. We aimed to study a SNAP-25 gene polymorphism, which is related to many psychiatric diseases, and FMS association in this prospective study.

**Methods:**

We included 71 patients who were diagnosed according to new criteria and 57 matched healthy women in this study. Both groups were evaluated regarding age, height, weight, BMI, education level, marital and occupational status. A new diagnosis of FMS was made from criteria scoring, SF-36, Beck depression scale, and VAS that were applied to the patient group. SNAP-25 gene polymorphism and disease activity score correlations were compared.

**Results:**

Mean age was 38±5,196 and 38.12±4.939 in patient and control groups, respectively (p=0.542). No significant difference was found between groups regarding age, height, weight, BMI, education level, marital or occupational status (p > 0.05). Ddel T/C genotype was significantly higher in the patient group (p = 0.009). MnlI gene polymorphism did not show a correlation with any score whereas a significant correlation was found between Ddel T/C genotype and Beck depression scale and VAS score (p < 0.05).

**Conclusion:**

FMS etiopathogenesis is not clearly known. Numerous neurologic, cognitive and psychological disorders were found during studies looking at cause. Our study showed increased SNAP-25 Ddel T/C genotype in FMS patients compared to the control group, which is related to behavioral symptoms, personality and psychological disorders in FMS patients.

## Background

Fibromyalgia syndrome (FMS) is a non-inflammatory rheumatologic disease characterized by widespread musculoskeletal pain, lethargy and tenderness without a definite cause [1]. FMS diagnosis is used for heterogeneous pathological states including anxiety disorders, depression, lethargy, sleep disorders, and gastrointestinal system symptoms with widespread pain [2]. One of the reasons that FMS is thought to be such a heterogeneous disease may be due to concurrent psychological disorders. Indeed, psychiatric symptoms are very common in this syndrome, and they influence the course of the disease [3,4]. FMS patients are reported to have increased depression, anxiety and bipolar disorder comorbidities [3,5]. Several studies determined disorders in personality inventory profiles based on the thought that some personality and mood disorders might predispose to FMS [6-8].

Dopamine is an important mediator in both psychopathological incidents and pain conduction. Dopamine D2 receptor sensitivity and density are increased in FMS patients [9]. Also, dopamine D4 receptor gene polymorphism is found to be relevant to FMS personality profile [10].

SNAP-25 protein is contributory to plasma membrane and synaptic vesicle [11]. SNAP-25 forms complexes with synaptobrevin in synaptic vesicles and with syntaxin in the plasma membrane [11]. In simple terms, SNAP-25 protein is critical in neurotransmission for fusion of plasma membrane and synaptic vesicle. Several studies investigated the relationship between SNAP-25 gene polymorphism and personality disorders, schizophrenia, and attention deficit and hyperactivity disorder; these studies reported that the SNAP-25 gene might influence development of these disorders [12-17]. Furthermore, SNAP-25 protein might be relevant to other different neurotransmitters due to its involvement in vesicle membrane transition and fusion. This is important because it can be the main reason behind neurological, cognitive, psychological disorders in FMS. If such a relationship exists, it will be enlightening for etiopathogenesis of FMS and treatment approaches. We aimed to evaluate the SNAP-25 gene (MnlI = rs3746544 and DdeI = rs1051312) polymorphism, which is related to many psychiatric diseases, and FMS association in this prospective study.

## Patients and methods

### Patients and evaluation

We included 71 female patients diagnosed with ACR 2010 fibromyalgia diagnosis criteria and 57 age-matched healthy females in the study. Patients and healthy volunteers were informed about the genetic evaluation and informed consent was taken. Blood (10 cc) was taken into EDTA tubes from both the patient and control groups and stored at -20°C. FMS new diagnosis criteria scoring (ACR 2010), VAS, SF-36, and Beck depression scale were applied to the patient group. SNAP-25 gene polymorphism prevalence and SNAP-25 polymorphism with disease activation association were compared. The Local ethics council of Pamukkale University Clinical Research Ethics Committee approved the study. Patient consent was obtained for this study.

### Tools

A sociodemographic information form was developed by the researchers and includes patient’s age, sex, education, socioeconomic status, settlement, marital status and disease period. The Beck depression scale (BDS) was used for determining the risk for depression, levels of depressive symptoms and difference of intensity. It was developed by Beck et al. and adapted to Turkish by Hisli (Hisli 1989). The VAS consists of 3 parts for measuring pain. The scale was adapted to Turkish and used in numerous studies. The SF-36 scale to determine quality of life was subjected to validity and reliability testing for its use in Turkish.

### Molecular analysis

Genetic evaluation was done in our university’s Medical Genetics Department. Patient and control genomic DNA was isolated from peripheral blood by a DNA Extraction Kit. The SNAP-25 genes MnlI (rs3746544) and DdeI (rs1051312) polymorphisms are on the 8^th^ exon (Forward 5′- TTC TCC TCC AAA TGC TGT CG-3′ and Reverse 5′- CCA CCG AGG AGA GAA AAT G-3′ primary series were used to replicate the UTR region).

In addition to these primer series, we also used 10X PCR Buffer, 5 μl dNTP mix consisting of 0.2 mM of every nucleotide and Taq polymerase enzyme. PCR reaction conditions were 95 degrees C for 2 minutes of denaturation followed by 95 degrees C for 45 seconds, 58 degrees C for 1 min, 72 degrees C for 2 minutes of 35 cycles and final elongation at 72 degrees C for 7 minutes.

10 U Ddel and 10 U MnlI enzymes were added separately to obtained 261 bp PCR products and this was incubated for 14 hours at 37°C for cutting. 3.5% Ultra pure agorose jelly was prepared for separating fragments after cutting. Later, PCR products were subjected to 40–50 minutes of electrophoresis and fragmented. After electrophoresis, the allele band series expected for Ddel polymorphism: for T allele: 261 bp cut band, for C allele: 228 bp and 33 bp two separate bands. The allele band series expected for MnlI polymorphism: for T allele: 256 bp and 5 bp two separate bands, for G allele: 210 bp, 46 bp and 5 bp three separate bands.

### Statistical analysis

For statistical analysis SPSS version 20 was used. Descriptive statistics were given as mean, standard deviation and percentage. Out study’s confidence interval was 95%. Intergroup significance was evaluated with Chi square test for qualitative data and Mann–Whitney U test for quantitative data. Quantitative data relationships were evaluated with Spearman correlation test. Non parametric variance analysis was done between genetic polymorphisms. p < 0.05 was considered as significant.

## Results

Mean age was 38±5,196 and 38.12±4.939 in patient and control groups, respectively (p=0.542). No significant difference was found between the groups regarding age, height, weight, BMI, education, settlement, marital or occupational status (p > 0.05) (Table 1). We determined MnlI gene polymorphism for TT genotype in 30 (42.3%), TG genotype in 34 (47.9%) and GG genotype in 7 (9.9%) patients. Control group had TT genotype in 27 (47.4%), TG genotype in 19 (33.3%) and GG genotype in 11 (19.3) patients. Dde1 genotype variance in patient group was as follows: TT genotype in 29 (40.8%), TC genotype in 40 (56.3%), TC genotype in 17 (29.8%) and CC genotype in 4 (7%). Intergroup SNAP-25 Mnl1 polymorphism variance was not significantly different (Table 2). Dde1 polymorphism variance between groups showed significant difference (p = 0.009). To determine the reason behind this significance, separate match evaluation was applied and TC genotype was discovered to be the reason. Patient and control groups Mnl1 and Dde1 polymorphism variance is given in Table 2.

**Table 1:** Demographic findings of patient and control groups

**Parameter**	**Patient (n=71)**	**Control (n=57)**	**p**
**Age**	38±5,196	38.12±4.939	0.542
**Number of labor**	2.01±0.902	1.96±0.566	0.721
**Number of children**	1.97±0.828	1.91±0.576	0.646
**Settlement n (%)**			
Urban	57 (80.3)	45 (75)	0.536
Rural	14 (19.7)	15 (25)	
**Marital status n (%)**			
Married	69 (97.2)	56 (98.25)	0.398
Single	2 (2.8)	1 (1.75)	
**Occupation n (%)**			
Working	21 (29.57)	17 (29.82)	0.703
Not working	50 (70.43)	40 (70.18)	

**Table 2:** Mnl1 and Dde1 polymorphism variance in patient and control groups

	**Patient n (%)**	**Control n (%)**	**p**
**Mnl1 gene**			
TT	30 (42.3)	27 (47.4)	0.149
TG	34 (47.9)	19 (33.3)	
GG	7 (9.9)	11 (19.3)	
**Dde1 gene**			
TT	29 (40.8)	36 (63.2)	**0.009**
TC	40 (56.3)	17 (29.8)	
CC	2 (2.8)	4 (7)	

We evaluated fibromyalgia new diagnosis criteria and their subscores, Beck depression scale, visual analogue scale (VAS), short form-36 (SF-36) subparameter scores (Table 3). Non parametric variance analyses between genetic polymorphisms were applied. When evaluated for Mnl1 polymorphism individuals for all three (TT, TG, GG) genotypes, VAS, BDS and SF-36 did not show significant differences (p > 0.05). When evaluated for Dde1 polymorphism individuals for all three (TT, TC, CC) genotypes, no significant difference was found for TT and CC individuals; however, TC genotype individuals were significantly higher for BDS (p = 0.045) and VAS scores (p = 0.033) (Table 4). This difference was significant after Bonferoni correction between the groups.

**Table 3:** Fibromyalgia new diagnostic criteria, VAS, Beck depression scale, SF-36 scores for patients

**Parameter**	**Score**
**Fibromyalgia new criteria**	
WPI1	15.27±1.80
Lethargy	2.28±0.565
Not rested	2.20±0.624
Cognition	1.82±0.661
Part 2a	6.35±1.43
Part 2b	1.99±0.521
**BDS**	16.77±10.638
**VAS**	85.04±12.629
**SF-36**	
Physical function	20.08±4.09
Physical role difficulty	4.97±1.25
Pain	5.33±1.33
General Health	11.25±4.01
Vitality	12.83±3.56
Social function	6.423±2.05
Emotional role	3.704±1.0197
Mental health	18.60±4.95

**Table 4:** VAS and BDI scores according to the presence TC genotypes

**Parameter**	**All patients**	**TC (+)**	**TC (-)**	**p**
BDS	16.77±10.6	17.6±11	15.18±10	**0.045**
VAS	85.04±12.629	86.41±12.14	83.14±11.83	**0.033**

## Discussion

The etiopathogenesis of FMS, which is characterized by widespread musculoskeletal pain and tenderness, is not clearly understood. FMS, chronic lethargy syndrome, irritable intestine syndrome, tension type headache, and myofacial pain syndrome are parts of central sensitization syndromes. Every one of these syndromes overlaps with FMS. One common feature of these syndromes is increased central neuron stimulation through various synaptic and neurotransmitter/neurochemical activities without structural pathology. This, however, is characterized by increased sensitivity to pressure and touch. Many factors contribute to central sensitization syndrome. These include pain, lethargy, sleep disorder, sensitivity to several stimuli, and psychosocial problems.

For the past several years, environmental as well as genetic conditions are blamed for FMS and related functional somatic disorders such as irritable intestine syndrome, migraine, and chronic lethargy syndrome [18-20]. Several studies tried to explain the etiopathogenesis of fibromyalgia syndrome. These studies showed that serum and central nervous system serotonin and its metabolite levels are low, and serotonin transport velocity in cerebrospinal fluid is slow. For this reason, the serotonin transport gene region has been brought to attention. Offenbaecher et al. reported that the S/S genotype is increased in FMS patients when compared to healthy subjects [21].

Dopamine is another molecule that is generating research interest, which has a role in both psychopathologic incidents and pain transport. Studies determined both dopamine levels and dopamine receptor gene polymorphisms and FMS [9,10,22,23].

It is known that psychiatric disorders, especially depression and anxiety disorder are quite common in FMS patients [3]. Several studies reported depression prevalence in FMS between 20% and 80% [3,4,7]. It is reported that FMS patients have difficulty in both understanding their own feelings and coping with emotions. Several studies determined disorders in personality inventory profiles based on the thought that some personality and mood disorders might predispose to FMS. When the personality inventory of patients was evaluated, hypochondriasis, hysteria, paranoia, and depression scales were found to be higher than control groups [6-8]. Studies indicated that low ability to cope with everyday problems can trigger FMS or can increase symptom intensity [6,8]. As a result, some psychiatric disorders are more prevalent in FMS, and some personality and mood features can predispose to FMS development.

Nevertheless, the relationship between these situations and FMS is not clearly known. Psychiatric disorders are increased in FMS and are relevant to symptom intensity [4,7]. Some personality and mood features can predispose to FMS, in addition to these features revealing themselves as a result of stress for coping with pain [24,25].

There can be common pathophysiological features between FMS and psychiatric disorders and personalities. Dopamine and serotonin mediated neurotransmitter transport can be among these common biological factors [23]. However, these disorders have some common risks such as exposure to difficult experience. These factors cause stress in the patient, and prolonged stress leads to cytokine secretion. Increased cytokines precipitate both psychiatric diseases and increased pain perception [26,27].

It is impossible to explain only one cause for FMS and the etiopathogenesis of these disorders. In addition, the relationship between concurrent disorders and FMS is not clearly known.

SNARE (**s**oluble **N**-ethylmaleimide-sensitive factor **a**ctivating protein **re**ceptor) proteins have a role in fusion between organelles, and organelles with plasma membrane in eukaryotic cells. SNAP-25 is a SNARE protein found in the plasma membrane. These proteins have important roles in electrical conduction in nerve cells. Nerve transporters are found in synaptic vesicles and sent by exocytosis to the other synapse. For this reason, SNAP-25 affects dopamine and other neurotransmitter secretions, and thus adds to FMS pathogenesis. Therefore, SNARE like proteins should be studied carefully, leading to better understanding of etiopathogenesis and treatment approaches. Also, the relationship between FMS and concurrent psychiatric disorders and personality features can be explained. For this purpose, we found increased SNAP-25 Dde1 TC gene polymorphism in FMS patients in this prospective study. In individuals with the TC gene genotype, BDS and VAS scores were found to be significantly higher than in individuals without TC genotype. FMS is one of the central sensitization syndromes. One mutual property of these diseases is that neurons have increased stimulus mediated by several synaptic and neurotransmitter/neurochemical activities without structural pathology. This results in increased reaction to basic stimuli. Patients who have the TC genotype have increased BSD and VAS scores in our study, which can result from different affected neurotransmitter functions both from this polymorphism and in pain transport. There are no studies done in this matter, and our results should be compared with similar studies.

The primary limitation of this study is trying to explain a complex disease such as FMS with only one mutation with a small number of patients. With more patients and other polymorphisms related to the disease, it will help us understand FMS better in the future.

## Conclusions

FMS etiopathogenesis is not clearly known. Numerous neurologic, cognitive and psychological disorders were found during studies attempting to identify cause. Our study showed increased SNAP-25 Ddel T/C genotype in patients compared to controls, which is related with behavioral symptoms, personality and psychological disorders in FMS patients.

## Competing interests

The authors declare that they have no competing interests.

## Authors’ contributions

AB: Study planning, organization, data collection, evaluation of results and article writing. CS: Evaluation of results, article writing and approval. HB: Creation of patient and control groups and obtaining informed consent, data collection and statistical analysis. ET: Molecular analysis of patient and control groups. VC (senior author): Article writing and approval. All authors read and approved the final manuscript.

## Pre-publication history

The pre-publication history for this paper can be accessed here:

http://www.biomedcentral.com/1471-2474/15/191/prepub
